# IS-CAT: Intensity–Spatial Cross-Attention Transformer for LiDAR-Based Place Recognition

**DOI:** 10.3390/s24020582

**Published:** 2024-01-17

**Authors:** Hyeong-Jun Joo, Jaeho Kim

**Affiliations:** 1 Department of Information and Communications Engineering, Sejong University, Seoul 05006, Republic of Korea; jhj7237.sejong@gmail.com; 2 Department of Electrical Engineering, Sejong University, Seoul 05006, Republic of Korea

**Keywords:** LiDAR place recognition, SLAM, cross-attention transformer network, IS-CAT

## Abstract

LiDAR place recognition is a crucial component of autonomous navigation, essential for loop closure in simultaneous localization and mapping (SLAM) systems. Notably, while camera-based methods struggle in fluctuating environments, such as weather or light, LiDAR demonstrates robustness against such challenges. This study introduces the intensity and spatial cross-attention transformer, which is a novel approach that utilizes LiDAR to generate global descriptors by fusing spatial and intensity data for enhanced place recognition. The proposed model leveraged a cross attention to a concatenation mechanism to process and integrate multi-layered LiDAR projections. Consequently, the previously unexplored synergy between spatial and intensity data was addressed. We demonstrated the performance of IS-CAT through extensive validation on the NCLT dataset. Additionally, we performed indoor evaluations on our Sejong indoor-5F dataset and demonstrated successful application to a 3D LiDAR SLAM system. Our findings highlight descriptors that demonstrate superior performance in various environments. This performance enhancement is evident in both indoor and outdoor settings, underscoring the practical effectiveness and advancements of our approach.

## 1. Introduction

Place recognition, a critical component in autonomous navigation systems, involves the re-identification of previously observed locations, essential for robots and self-driving vehicles navigating within specific environments. This capability is particularly essential in simultaneous localization and mapping (SLAM) systems for loop closure detection, an area with widespread applicability in diverse domains.

Camera-based visual place recognition methods [1,2,3,4] are advantageous due to their ability to utilize detailed visual information for specific place recognition and cost-effective implementation. However, these methods are plagued by problems related to adapting to changing environmental conditions. This has led to a surge in research focusing on LiDAR’s robustness, particularly against changes such as weather variations and the differences in lighting between day and night. Recent studies have primarily explored the creation of global descriptors from LiDAR data for localizing robots in large outdoor spaces, typically utilizing unordered 3D point clouds [5,6,7,8] to capture detailed spatial information. In addition, significant research on transforming complex 3D LiDAR data into more manageable 2D projections, such as spherical and bird’s eye views (BEV), has provided unique spatial insights [9,10,11,12,13,14,15]. This strategic shift to 2D mode has maintained the richness of LiDAR data while processing the data more efficiently. Notably, recent advances include hybrid models that have blended multiple 2D perspectives for a fuller spatial understanding [16] or combined point-wise and voxel-wise features from 3D point clouds to enhance place recognition accuracy [17]. The acquisition of spatial data via LiDAR is critical in capturing environmental complexity, as it provides 3D point cloud representations enriched with measurable attributes such as distance and height. However, an over-reliance on the spatial data of LiDAR can result in challenges in environments characterized by uniform spatial structures, such as the long hallways commonly found in indoor settings. Consequently, the efficacy of place recognition tasks is often diminished due to the absence of distinct spatial features. To address this limitation, certain LiDAR studies exploit LiDAR intensity data, which aid in describing different objects based on the reflection of light, thereby aiding in the recognition of places in different settings, such as indoor environments [14,18,19,20]. Therefore, both spatial and intensity information derived from LiDAR data are vital to the recognition and understanding of environmental contexts. Considering their significance, there is a need for integrated research that leverages the strengths of each type of data and addresses their individual limitations. However, despite the potential of these approaches, there is a considerable lack of research that integrates LiDAR intensity and spatial data. Such integrative research facilitates the effective utilization of spatial data for capturing the physical dimensions of the environment and intensity data to discern the unique reflective properties of various objects, thereby enriching the overall environmental analysis.

Thus, this study proposes a novel intensity and spatial cross-attention transformer (IS-CAT), which is skillfully fabricated to encode and synergize spatial (depth and height) and intensity data from two unique LiDAR perspectives: range view and bird’s eye view. The methodology initiates by generating I-S images that encode spatial and intensity information in spherical and top-down projections, each displayed in multi-layered structures. These data segments are then processed through a conv-transformer sequence. Consequently, separate volumes of spatial and intensity features are created. Finally, a cross-attention transformer-to-concatenation module interlinks these diverse insights, effectively uncovering important correlations and generating a robust global descriptor for accurate place recognition.

The primary contributions of this study are manifold. First, we propose an end-to-end network architecture designed to improve long-term place recognition significantly by leveraging the spatial and intensity information of complex LiDAR data and condensing them into efficient descriptors. To the best of our knowledge, this study is the first to generate a global descriptor that understands the correlation between spatial and intensity from extensive LiDAR data. We validate with a variety of experiments the performance of our approach using the extensive NCLT dataset and our own dataset, the Sejong indoor-5F dataset. Lastly, we successfully integrate this technology into the SLAM system as a loop closure module. The approach is further demonstrated by its application to the Sejong indoor-5F dataset, which encompasses a featureless indoor environment within a building at Sejong University.

## 2. Related Work

In this section, we examine the prior studies that lay the foundation for our proposed intensity and spatial cross-attention transformer (IS-CAT). We categorize the existing research into two primary streams: hand-crafted and learning-based descriptors, emphasizing the evolution from traditional techniques to more advanced, learning approaches.

### 2.1. Hand-Crafted Descriptors

Early attempts at place recognition leveraged hand-crafted descriptors, which are methods wherein features are manually designed and extracted from sensor data. Although often reliant on the expertise and assumptions of the researchers, these methods have been vital to the advancement of our initial understanding; however, they have certain limitations.

Geometry-based methods: most of these methods begin by performing a 2D projection of LiDAR data to encode geometric information. Scan context (SC) [9], a pioneering work on the representative BEV method, performs encoding by maintaining the maximum height of the point and dividing the horizontal space into individual bins to generate a 2D matrix descriptor. SC has exhibited excellent performance in urban environments. Thereafter, several studies have been conducted based on SC-based transformation methods, extending them to various areas [18,19,20,21,22]. RID [10] is a spherical projection method that generates a descriptor that compresses distance information to increase performance and search speed in an indistinguishable environment. ESF [23] suggests a shape descriptor that integrates angle, point distance, and area to enhance recognition capabilities. Meanwhile, M2DP [24] focuses on projecting a point cloud onto various 2D planes, creating concise signatures. These signatures undergo singular value decomposition (SVD), and the initial left and right singular vectors from this process are merged to form the M2DP descriptor. LiDAR Iris [25] obtains a binary signature image from a point cloud and extracts additional features based on LoG-Gabor filtering.

Intensity-based methods: certain studies have used the intensity information inherent in LiDAR data to exploit how different materials reflect light differently to distinguish between environments. The intensity scan context (ISC) [18] was designed to perform well in indoor environments; and was created using intensity information instead of maximum height. WSC [19] and FSC [20] fuse intensity information and other information (e.g., geometry, density) to create a global descriptor that can complement SC and ISC. DELIGHT [26] generate a total of 16 non-overlapping bins from two spheres with horizontal and azimuthal splits. They compute an intensity histogram for each bin and perform global localization via keypoint-based geometry verification.

Overall, while hand-crafted descriptors benefit from not needing retraining, their performance is capped, and they do not benefit from additional data in terms of improvement.

### 2.2. Learning-Based Descriptors

3D point-cloud-based methods: these methods employ neural networks to process raw, unordered 3D point clouds directly, learning robust feature representations. PointNetVLAD [5] leverages the PointNet [27] framework and NetVLAD [2] for end-to-end training, thus focusing on extracting comprehensive global descriptors. Furthermore, PCAN [6] analyzes the use of an attention mechanism specifically for aggregating local features. MinkLoc3D [8] uses sparse 3D convolutions to identify crucial features within the point cloud, facilitating accurate localization. It incorporates generalized-mean (GeM) pooling [28] for the effective calculation of a condensed global descriptor. Although powerful, these methods often required substantial computational resources and were plagued with data inefficiency issues. NDT-Transformer [29] marked a pioneering step in applying transformer networks to point cloud place recognition. This model uniquely transforms point cloud data into normal distribution transformation units, utilizing a series of encoders for feature integration. The network then applies the NetVLAD layer, effectively creating robust global descriptors for the recognition process. SOE-Net [7] introduces a new approach for point cloud-based place recognition, combining point orientation encoding with a self-attention mechanism. This method enhances global descriptor extraction, leading to improved performance in large-scale environments. SVT-Net [30] incorporates sparse voxel transformers, specifically the atomic-based and cluster-based versions, to learn short-range local features and long-range contextual features in efficiently processing large-scale data.

Projection-based methods: recognizing the computational challenges of processing 3D data, several studies proposed the conversion of LiDAR scans into 2D projections (e.g., range views, BEV) as inputs for convolutional neural networks. A scan context image (SCI) [31] enhances the original SC [9] by incorporating a three-channel system, which facilitates robot localization on grid maps using a convolutional neural network for place classification. OverlapNet [12] is a comprehensive deep neural network that integrates various data types, including intensity, normals, and semantics, with LiDAR range images, thus facilitating the comparison of 3D scans. Furthermore, OverlapTransformer [13] applies the transformer module to range images and creates a yaw-angle invariant descriptor to ensure complete operation in the opposite direction. However, these approaches sometimes oversimplified the spatial relationships or lost critical 3D information during the projection process. SeqOT [15] employs a transformer network for LiDAR-based place recognition, using spatial–temporal data from sequential images. Its efficient and robust approach provides improved performance in diverse environments and against viewpoint changes.

Hybrid-based methods: more recent efforts have attempted to combine multiple types of data or use different data modalities to generate richer and more reliable descriptors. These hybrid methods, with geometric or spatial data, can enhance recognition accuracy across varied conditions. FusionVLAD [32] introduced a network architecture that operates in parallel, encoding both spherical and top-down projections simultaneously. They focus on synthesizing data from multiple perspectives, aiming to create an integrated perspective by combining information from these diverse views. In addition, CVTNet [16] goes beyond simply encoding spherical and top-down projections. It splits the data into multiple layers, enhancing spatial information. An inter-transformer is employed to capture the relationship between these two perspectives effectively. CASSPR [17] designed a network to overcome the shortcomings of point- and voxel-wise features by utilizing a hierarchical cross-attention transformer.

Despite the significant progress in learning-based descriptors, the simultaneous utilization of spatial and intensity information within suitable models for place recognition remains unexplored. Existing studies have either treated these elements in isolation or have not fully utilized their synergistic potential. This research void drives our proposed IS-CAT model, designed to integrate these data types, interlinking their combined strengths while helping their respective shortcomings.

In this study, we introduce a novel transformer network that combines multi-perspective views and diverse spatial and intensity data derived from LiDAR. By reinforcing correlations between spatially coherent features and amplifying the associations with environmental objects based on intensity data, our model constructs a comprehensive global descriptor.

## 3. Proposed Approach

**Figure 1:**
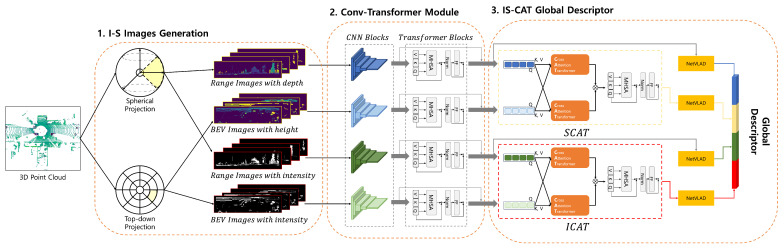
Overview of the IS-CAT model for place recognition. The process begins with the generation of I-S images that encode spatial and intensity data from LiDAR inputs, using interval-based splitting for distance and height, and applying spherical and top-down projections. These images are then processed through a conv-transformer module, producing four feature volumes, two each for spatial and intensity features. Finally, a cross-attention transformer module synthesizes these features into a 1D global descriptor for robust place recognition, with dedicated submodules for spatial and intensity data integration. Spatial information is depicted in yellow and intensity information in red to facilitate differentiation.

In this section, we introduce a proposal for the overall process of our IS-CAT model, which is designed to recognize places, as illustrated in Figure 1. Our approach comprises three main parts: I-S images generation, conv-transformer module, and IS-CAT global descriptor. The initial step involves generating I-S images, which effectively encode both spatial and intensity information extracted from LiDAR data. I-S images incorporate techniques for splitting intervals based on distance and height, partitioning images using spherical and top-down projection methods from each interval, and encoding spatial and intensity information. Subsequently, the generated I-S images are processed through the conv-transformer module, which comprises convolution and transformer layers. Each conv-transformer module outputs four distinct feature volumes: two each for spatial and intensity features. Finally, the IS-CAT global descriptor is generated from the spatial and intensity feature volumes. This process utilizes a cross-attention transformer-to-concatenation module. It contains two submodules: one focusing on spatial and the other on intensity.

This module outputs a 1D descriptor that captures both spatial and intensity information from the input I-S images. This structured approach facilitates effective encoding and synergizing of spatial and intensity data from LiDAR data, thus enabling robust place recognition in challenging environments.

### 3.1. I-S Images Generation

**Figure 2:**
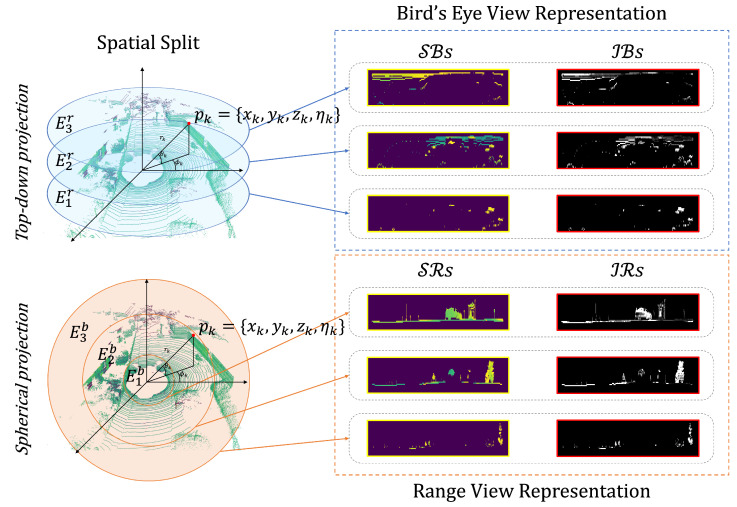
Method for generating intensity-spatial (I-S) images through from LiDAR data via 2D projection after spatial segmentation. It shows the top-down and spherical views, dividing the point cloud into layers for bird’s eye (depicted in blue) and range (depicted in orange) view representations.

The generation of intensity-spatial (I-S) images from LiDAR data is critical in our research, facilitating enhanced place recognition. These images are unique in their capacity to convey the spatial arrangement and the intensity variations of the LiDAR data simultaneously, providing a multifaceted understanding of the scanned area. We categorize the LiDAR data by both the distance and elevation of objects, considering different perspectives to capture comprehensive details. The creation of I-S images employs both traditional spherical and top-down projection techniques. Inspired by the approach used in [16], we segment the space based on distance and elevation. We produce I-S images that record spatial division and integrate spatial and intensity information through range view and bird’s eye view representations, as illustrated in Figure 2.

To partition the space, we use two parameters: distance and height. Spherical and top-down representations are derived from distance information and height data, respectively. The spatial segments based on distance are defined as $E_{r}=\left\{E_{1}^{r},E_{2}^{r},\ldots ,E_{q}^{r}\right\}$. Likewise, the spatial segments based on height are defined as $E_{b}=\left\{E_{1}^{b},E_{2}^{b},\ldots ,E_{q}^{b}\right\}$.

From these spatial segments, we derive a quartet of images that encapsulate distinct aspects of the space: SR (spatial range) and IR (intensity range), which are generated through the range view representation, and SB (spatial bird’s eye) and IB (intensity bird’s eye), which are products of the bird’s eye view representation.

Denote the intensity reading as η. A LiDAR scan can be represented as a set, $P=\left\{p_{1},p_{2},\cdots ,p_{n}\right\}$, when there are n points, the expression for *k*: $k\in \left\{\begin{array}{c} 1,n \end{array}\right\}$, where each point, $p_{k}$, is a Cartesian coordinate $\left\{x_{k},y_{k},z_{k},\eta_{k}\right\}$ that is transformed to a polar coordinate through Equation (1):

(1) $$\left\{\begin{array}{c} r_{k}=\sqrt{x_{k}^{2}+y_{k}^{2}+z_{k}^{2}}\\ \phi_{k}=\arctan\frac{x_{k}}{y_{k}}\\ \theta_{k}=\arcsin\frac{y_{k}}{r_{k}} \end{array}\right.$$

where $r_{k}$ denotes the Euclidean distance of the point $p_{k}$ from the origin, $\phi_{k}$ denotes the azimuth angle, and $\theta_{k}$ denotes the elevation angle.

The range view images follow the mathematical representation outlined below, with Equation (2) defining the projection of LiDAR points onto the image plane:

(2) $$\left[\begin{array}{c} u_{k}^{r}\\ v_{k}^{r} \end{array}\right]=\left[\begin{array}{c} \frac{1}{2}\left[\phi_{k}\pi^{-1}+1\right]W\\ \left[1-\left(\theta_{k}+\left|f_{\mathrm{down}\;}\right|\right)f^{-1}\right]H \end{array}\right],$$

where $u_{k}^{r}$ and $v_{k}^{r}$ indicate the pixel coordinates within the image, *W* and *H* denote the width and height of the range image, respectively, *f* denotes the vertical field of view, and $f_{up}$ and $f_{down}$ denote the upper and lower limits of the field of view, respectively. By transforming the 3D LiDAR point cloud data into a 2D image, the data can be represented visually.

For the *p*-th spatial range image, denoted as ${\mathrm{SR}}_{p}$, we employ Equation (3) to establish the maximum distance value within each spatial segment, $E_{p}^{r}$:

(3) $${\mathrm{SR}}_{p}^{ij}=\max_{k=1}^{N_{ij}}\left\{r_{k}\right\},\;\mathrm{where}\;N_{ij}=\left|\left\{k|\left(u_{k}^{r},v_{k}^{r}\right)\in P_{ij}\right\}\right|.$$

Equation (3) computes the farthest distance, ${\mathrm{SR}}_{p}^{ij}$, for each cell (i,j) within the image that corresponds to the space $E_{p}^{r}$, and $N_{ij}$ is the number of points within that cell. Thus, ${\mathrm{SR}}_{p}^{ij}$ represents the longest distance measured from all the points that are located within the cell (i,j) of the *p*-th image.

Similarly, for the *p*-th intensity range image, represented as ${\mathrm{IR}}_{p}$, we use analogous Equation (4) to identify the maximum intensity value within the same spatial segment $E_{p}^{r}$:

(4) $${\mathrm{IR}}_{p}^{ij}=\max_{k=1}^{N_{ij}}\left\{\eta_{k}\right\},\;\mathrm{where}\;N_{ij}=\left|\left\{k|\left(u_{k}^{r},v_{k}^{r}\right)\in P_{ij}\right\}\right|$$

where ${\mathrm{IR}}_{p}^{ij}$ signifies the highest intensity value among all the points within the cell (i,j) of the *p*-th image, which also falls under the domain of the space $E_{p}^{r}$.

For the bird’s eye view representation, the projection of LiDAR points onto a 2D plane is described by Equation (5):

(5) $$\left[\begin{array}{c} u_{k}^{b}\\ v_{k}^{b} \end{array}\right]=\left[\begin{array}{c} \frac{1}{2}\left[\phi_{k}\pi^{-1}+1\right]W\\ \left[r_{k}f^{-1}\right]H \end{array}\right].$$

We generate the *p*-th spatial bird’s eye image, ${\mathrm{SB}}_{p}$, and the *p*-th intensity bird’s eye image, ${\mathrm{IB}}_{p}$, using similar principles as those applied to the range view images but adjusted for the BEV perspective. The spatial bird’s eye image, ${\mathrm{SB}}_{p}$, is computed to represent the maximum height values within the space $E_{p}^{b}$:

(6) $${\mathrm{SB}}_{p}^{ij}=\max_{k=1}^{N_{ij}}\left\{z_{k}\right\},\;\mathrm{where}\;N_{ij}=\left|\left\{k|\left(u_{k}^{b},v_{k}^{b}\right)\in P_{ij}\right\}\right|.$$

Similarly, the intensity bird’s eye image, ${\mathrm{IB}}_{p}$, reflects the maximum intensity values corresponding to the same spatial segment $E_{p}^{b}$:

(7) $${\mathrm{IB}}_{p}^{ij}=\max_{k=1}^{N_{ij}}\left\{\eta_{k}\right\},\;\mathrm{where}\;N_{ij}=\left|\left\{k|\left(u_{k}^{b},v_{k}^{b}\right)\in P_{ij}\right\}\right|.$$

Therefore, each *p*-th BEV image conveys unique spatial information from a top-down perspective, with ${\mathrm{SB}}_{p}$ highlighting the structural layout and ${\mathrm{IB}}_{p}$ emphasizing the reflective properties from above.

Through the adoption of the methodologies outlined above, we have systematically partitioned the spatial domain and subsequently generated four distinct sets of images: the spatial range (SRs) and intensity range (IRs) images from the range view, as well as the spatial bird’s eye (SBs) and intensity bird’s eye (IBs) images from the bird’s eye view. These images, which are derived from conventional projection methods, are rich with both spatial and intensity information. They are designed to serve as integral inputs for our model, providing it with the detailed data required to perform place recognition tasks effectively. The dual perspectives of the range and bird’s eye views ensure a comprehensive understanding of the environment, facilitating a robust input dataset for the subsequent processing stages.

### 3.2. Conv-Transformer Module

After extracting the four distinct image sets, we enhance shape recognition capabilities by combining convolutional neural networks (CNNs) and transformer modules, utilizing complex spatial and intensity information. Each image set, structured as H×W×D, aligns with the OverlapNetLeg CNN architecture employed in [12]. These sets are processed by a quartet of CNNs, including a seven-layer deep fully convolutional network (FCN) leg that preserves yaw-angle-equivariant, as demonstrated in [13,16]. With W=900 and H=32, the leg outputs a compact feature volume of 1×900×256.

To refine feature discrimination further, we employ a transformer encoder equipped with a multi-head self-attention (MHSA) mechanism that effectively captures complex inter-feature relationships, enhancing image representation. The attention mechanism applied to the SR image features is formally described by:

(8) $$\begin{array}{cc} A^{SR} & =\mathrm{Attention}\left(Q^{SR},K^{SR},V^{SR}\right)\\ \; & =\mathrm{softmax}\left(\frac{Q^{SR}K^{SR^{T}}}{\sqrt{d_{k}}}\right)V^{SR} \end{array}.$$

In Equation (8), $Q^{SR},K^{SR}$, and $V^{SR}$ denote the queries, keys, and values extracted from the convolutional legs, with $d_{k}$ representing the key’s dimensionality. The attention-enriched feature maps, $A^{SR}$, are then passed through a feed-forward network (FFN) coupled with layer normalization (LN), resulting in the refined feature volume, $S^{SR}$, as follows:

(9) $$\begin{array}{cc} S^{\mathrm{SR}} & =\mathrm{LN}(\mathrm{FFN}\left(\mathrm{LN}(C\left(F^{\mathrm{SR}},A^{\mathrm{SR}}\right))\right)\\ \; & \;+\mathrm{LN}(C\left(F^{\mathrm{SR}},A^{\mathrm{SR}}\right))) \end{array}$$

where C(·) is the concatenation operation along the channel dimension. This procedure transforms a coarse feature volume, $F^{SR}$, extracted from the convolutional legs, into an attention-enhanced representation, $S^{SR}$, which is subsequently used for downstream applications. The aforementioned process, initially applied to SR images, is identically applied to all other image sets, ensuring a uniform approach to feature enhancement across the entire spectrum of I-S images.

With prior research from [13,15,16], our method integrates the concept of yaw-angle-equivariant into feature extraction. We base our approach on this research’s findings and demonstrate that our features also maintain this important characteristic. Specifically, we utilize the FCN leg from the OverlapNetLeg CNN architecture, which is designed to be yaw-angle-equivariant. By applying our transformer encoder to the FCN leg’s output, we ensure that the resulting features remain consistent, even when the yaw angle changes. The attention mechanism in our model also considers yaw-angle-equivariant. Consequently, features are better suited for shape recognition, as they robustly capture the spatial relationships while remaining insensitive to rotations around the vertical axis. Therefore, the proposed model’s features maintain a yaw-angle-equivariant, which facilitates good performance in place recognition.

### 3.3. IS-CAT Global Descriptor

**Figure 3:**
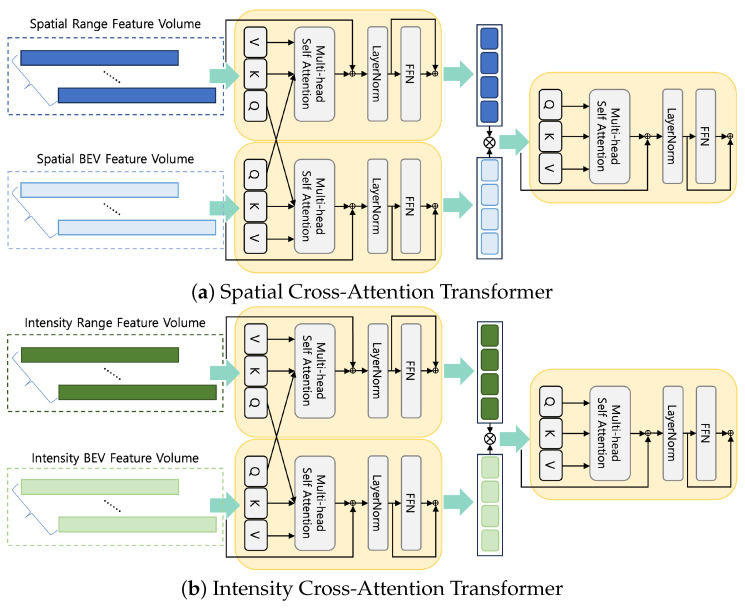
The IS-CAT module in two distinct configurations. Each module processes corresponding feature volumes to enhance feature representation with cross attention to concatenation. The spatial module (**a**) focuses on the BEV and range spatial features, while the intensity module (**b**) emphasizes intensity features from both BEV and range data.

Finally, our method concludes with the generation of IS-CAT global descriptors designed to facilitate place recognition. We propose spatial cross-attention transformer (SCAT) and intensity cross-attention transformer (ICAT) techniques to capture inter-correlations of spatial and intensity, vital to obtaining an environmental understanding. The complexity of SCAT and ICAT is illustrated in Figure 3.

Cross attention is crucial for mapping the interrelationship between distance and height information, allowing a deeper understanding of the spatial layout within the surrounding environment. Recognition of environmental features is not solely dependent on spatial data. The material properties of an object also play an important role. This is particularly true when distinguishing among features such as buildings or signs in structurally similar environments, including indoor environments, where material information can offer unique clues. Therefore, ICAT aims to generate powerful features that can identify object properties and materials from spatially segmented image data, providing a more robust perception of the environment. Furthermore, we adopted the cross-attention-to-concatenation method. This method facilitates the modeling of the global context with improved granularity by connecting the two streams of cross attention and processing them through an additional transformer. This hierarchical approach to cross-modal interaction has been thoroughly investigated in [33], and helps alleviate limitations associated with existing cross-attention mechanisms. Our implementation achieves a deeper understanding of complex data interactions and performs rich, layered interactions.

The mathematical formulation for SCAT, operating on spatial feature volumes denoted as $S^{S}$, is defined as follows:

(10) $$\left\{\begin{array}{c} Z_{\left(\mathrm{SR}\right)}\leftarrow MHSA\left(Q^{S^{\mathrm{SB}}},K^{S^{\mathrm{SR}}},V^{S^{\mathrm{SR}}}\right)\\ Z_{\left(\mathrm{SB}\right)}\leftarrow MHSA\left(Q^{S^{\mathrm{SR}}},K^{S^{\mathrm{SB}}},V^{S^{\mathrm{SB}}}\right)\\ Z_{\left(S\right)}\leftarrow Tf\left(C\left(Z_{\left(\mathrm{SR}\right)},Z_{\left(\mathrm{SB}\right)}\right)\right) \end{array}\right.$$

Multi-head self-attention (MHSA) facilitates the model’s ability to attend to information jointly across multiple representational subspaces. The query, $Q^{S^{\mathrm{SB}}}$, spatial bird’s eye view feature volume interacts with the key, $K^{S^{\mathrm{SR}}}$, and value, $V^{S^{\mathrm{SR}}}$, of the spatial range view feature volume. This enables the model to cross-reference the elevation and distance information. In the opposite case, $Q^{S^{\mathrm{SR}}}$, the same process is followed to create features for each $Z_{\left(\mathrm{SR}\right)}$ and $Z_{\left(\mathrm{SB}\right)}$. Transformer functions, denoted by Tf(·), represent the processing through successive transformer layers or blocks, thereby facilitating the sophisticated interpretation of spatial data. $Z_{\left(S\right)}$ represents a set of combined features after transformation that encapsulates spatial information and represents the comprehensive environmental characteristics we wish to capture.

Meanwhile, the mathematical formula of ICAT operating on the intensity feature volume denoted by $S^{I}$ is defined as:

(11) $$\left\{\begin{array}{c} Z_{\left(\mathrm{IR}\right)}\leftarrow MHSA\left(Q^{S^{\mathrm{IB}}},K^{S^{\mathrm{IR}}},V^{S^{\mathrm{IR}}}\right)\\ Z_{\left(\mathrm{IB}\right)}\leftarrow MHSA\left(Q^{S^{\mathrm{IR}}},K^{S^{\mathrm{IB}}},V^{S^{\mathrm{IB}}}\right)\\ Z_{\left(I\right)}\leftarrow Tf\left(C\left(Z_{\left(\mathrm{IR}\right)},Z_{\left(\mathrm{IB}\right)}\right)\right) \end{array}\right.$$

It follows a hierarchical structure similar to that of SCAT. Further, it improves the model’s ability to understand object properties and materials, which are important aspects when distinguishing among environmental features.

In the final stage of our model, spatial, Z(S), and intensity, Z(I), feature sets undergo the NetVLAD-MLPs combos, resulting in the generation of the IS-CAT’s global descriptors $g_{i}$ and $g_{s}$, respectively. Moreover, in accordance with our full pipeline, NetVLAD-MLPs combos are also applied to $S^{\mathrm{SR}}$ and $S^{\mathrm{IR}}$, yielding supplementary descriptors $g_{sr}$ and $g_{ir}$. This process culminates in the formation of a composite descriptor [$g_{i}$, $g_{s}$, $g_{sr}$, $g_{ir}$]. The reasoning for this design is detailed in the results discussed in Section 4.3. For network training, we followed the methodology proposed by [12] that focuses on the overlap between scan pairs by adopting a lazy triplet loss. We set our parameters to treat overlaps greater than 0.3 as positive samples, and those that do not satisfy this threshold as negative samples. Furthermore, we maintain the existing parameters for the triplet loss with $k_{p}=6$, $k_{n}=7$, and a margin α=0.5 to avoid negative loss.

**Figure 4:**
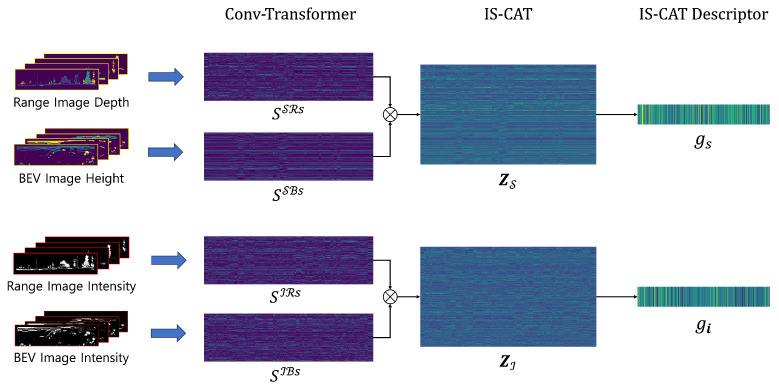
Feature extraction visualization from the IS-CAT network. This figure showcases the process from raw image inputs through conv-transformer layers, leading to the IS-CAT module, and resulting in the final IS-CAT descriptor. It highlights how each layer processes and describes different features, reflecting their varying perspective and data types while building global descriptors.

The distinction of each feature through this approach is presented in Figure 4. The features extracted at each layer post the initial conv-transformer module processing are visualized. This visualization enables discerning between the features arising from spatial and intensity information. Notably, even within the same range view representations, the features extracted based on depth and intensity values manifest distinct patterns. This variance highlights the benefits of integrating diverse spatial and material property data to gain environmental understanding. Further analysis of the IS-CAT module reveals the distinct nature of SCAT and ICAT features. SCAT focuses on exhibiting the connection among different views in terms of space through features, whereas ICAT reveals both the spatial correlations and object reflectivity, thereby contributing to material property and surface attributes. These insights affirm the distinctiveness of each feature set, a diversity that is mirrored in the variety of descriptors produced, as shown in Figure 4.

## 4. Experiments

In this section, we evaluate the experimental results to validate the effectiveness of the proposed method. Two datasets are considered: NCLT, covering a wide range of indoor and outdoor environments, and our own Sejong indoor-5F dataset collected by the AGV. Our evaluation begins with an analysis of different approaches to cross-attention transformer (CAT) and LiDAR data, focusing on their critical information and perspectives to explain our model’s design. We also demonstrate that our method achieves state-of-the-art performance in specific NCLT dataset sequences, highlighting its effectiveness across varied environments. Finally, we test our model on the Sejong indoor-5F dataset in feature-limited indoor areas, such as hallways, proving its ability to maintain map consistency in a real SLAM system and its practical applicability in actual settings.

### 4.1. Experimental Setup

#### 4.1.1. Datasets

**Figure 5:**
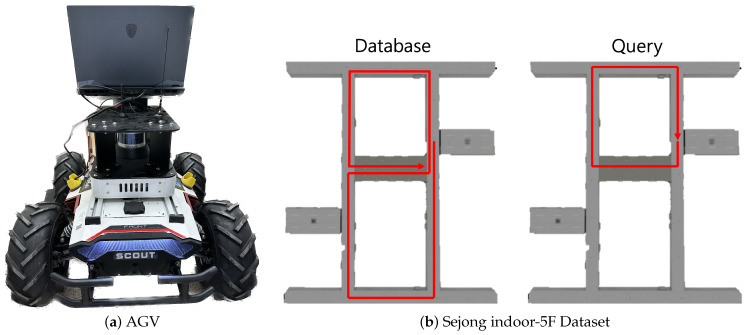
Data acquisition for Sejong indoor-5F dataset. (**a**) Showcases the AGV equipped with a LiDAR system, used for indoor data collection. (**b**) Presents the Sejong indoor-5F dataset, containing the database and query sections within the building’s layout.

The NCLT dataset [34] is a collection of long-term measurements obtained via a 32-ray LiDAR (Velodyne HDL-32E (San Jose, CA, USA)) system mounted on a Segway mobile platform covering similar routes on different days. The data were gathered through repeated explorations of the campus, both indoors and outdoors, on different paths, at different times of the day, and during various seasons. The Sejong indoor-5F dataset was collected via an AGV, as shown in Figure 5, which used LiDAR (Velodyne VLP-16) and was equipped with an edge device (intel i9-12900HK, RTX 3080 Ti 16G (Santa Clara, CA, USA)) to collect data. Sejong indoor-5F is an indoor environment, such as a featureless long hallway, and single point cloud scan data were gathered at approximately 1-m intervals. Both hallways were approximately similar in shape, with a large hallway and elevator in the center. To create a loop, data were collected through a total of two rotations, and the collected data were divided into a query and database to enable place recognition.

#### 4.1.2. Implementations

In the NCLT and Sejong indoor-5F datasets, the I-S image set size was set to 32×900. For processing these images, we segmented the range into 0 to 60 m intervals. Subsequently, we concatenated a four-layer image with another image that encompassed the entire range from each range view image. Similarly, for the bird’s eye view images, we compiled images containing four layers and the full height range, spaced at intervals of −4 to 12 m, ensuring uniform input dimensions. This approach resulted in a total input size of 4×5×32×900 for each set of four images. Additionally, the architecture of IS-CAT incorporates module parameters derived from [16]. Table 1 details the parameters employed in our study. The term $d_{model_{b}}$ represents the embedding dimension prior to concatenation, while $d_{model_{a}}$ indicates the embedding dimension post-concatenation. The output descriptor size is 1×1024. To assess the performance of our proposed model, IS-CAT, we conducted a comparative analysis against both hand-crafted and learning-based methods. For hand-crafted methods, we utilized SC [9], ISC [18], M2DP [24], and RID [10]. Except for M2DP, which has a descriptor size of 1×192, all other hand-crafted methods employ a descriptor size of 20×60. Regarding the learning-based methods, the majority are structured with a descriptor size of 1×256. However, CVTNet [16] and FusionVLAD [32] are exceptions, designed with descriptor sizes of 1×768 and 1×2048, respectively.

To integrate into the 3D LiDAR SLAM system, we adopted the A-LOAM (https://github.com/HKUST-Aerial-Robotics/A-LOAM, accessed on 18 August 2022) method, known for its user-friendly approach to LiDAR odometry. The implementation was executed in C++ and was specifically architected to conduct pose-graph optimization, utilizing GTSAM [35] as the solver, referring to the approach in the SC-A-LOAM (https://github.com/gisbi-kim/SC-A-LOAM, accessed on 10 January 2023) method.

### 4.2. Analysis of Cross-Attention Transformer

**Figure 6:**
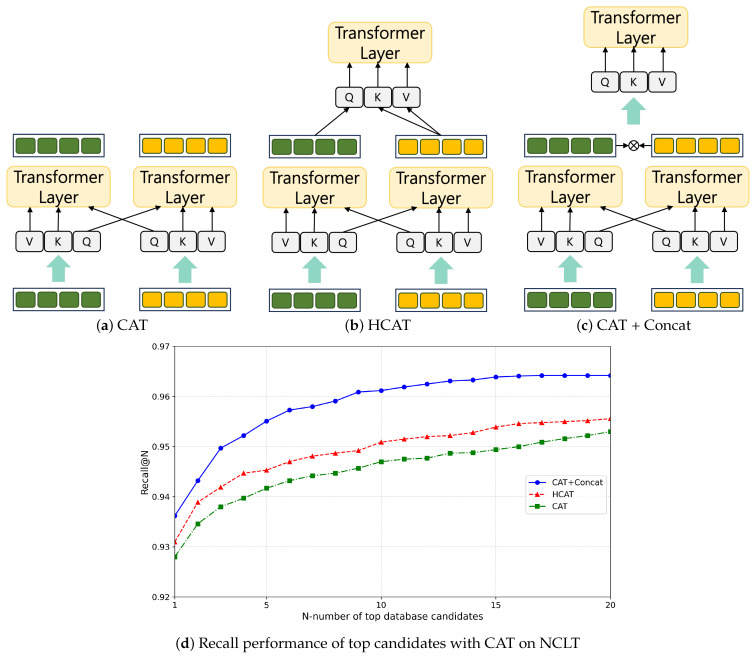
Analysis of CAT modules to design IS-CAT.

The first experiment investigated various cross-attention transformer (CAT) methods. To evaluate the model, training was performed on the 2012-01-08 sequence of the NCLT dataset, while testing was conducted on the 5 February 2012 sequence. We used average recall AR@N as the metric of the evaluation. In [33], it was shown that, to perform CAT, the embedding of Q (query) must be exchanged for cross-modality interaction, as shown in Figure 6a. CASSPR [17] proposed a hierarchical cross-attention transformer (HCAT) to learn between point-wise and voxel-wise features. As shown in Figure 6b, the information considered more important was input as Q, and the remaining information was input as K and V. Finally, the CAT was introduced to the concatenation method presented in [33]. As shown in Figure 6c, after performing CAT, concatenation was performed on each feature, followed by attention being performed on this feature once more to strengthen the feature. This method complemented the shortcomings of the existing CAT and facilitated the learning of richer features. The results are shown in Figure 6d. As evident, when designing IS-CAT, the CAT-to-concatenation method exhibited the best performance by compensating for the shortcomings of the existing CAT. Next, it was confirmed that the performance results were obtained using the HCAT and CAT methods.

### 4.3. Analysis of LiDAR Representation and Data

**Figure 7:**
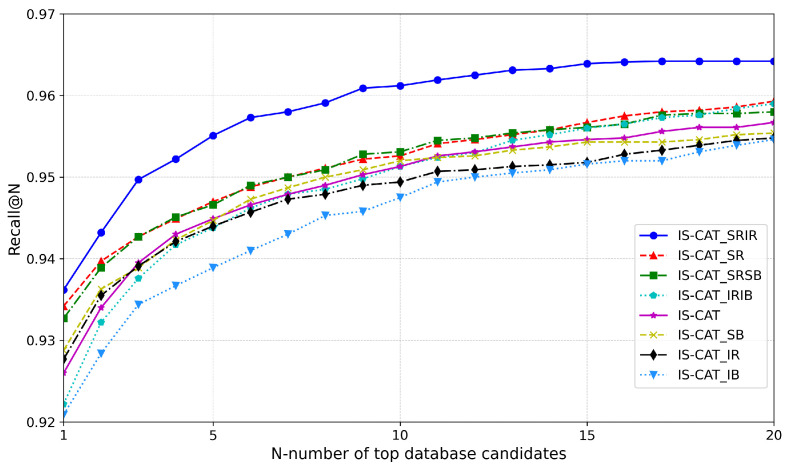
Analysis of important representation and information from LiDAR.

Following the development of the IS-CAT model, we conducted an experiment focusing on generating descriptors. This was achieved by manipulating various views, along with spatial and intensity information in 2D representations. We performed training on the 8 January 2012 sequence of the NCLT dataset, whereas the evaluation was performed on 5 February 2012. Initially, the standard descriptor produced by IS-CAT was employed. Simultaneously, we generated additional descriptors based on four types of information: $S^{\mathrm{SR}}$, $S^{\mathrm{SB}}$, $S^{\mathrm{IR}}$, and $S^{\mathrm{IB}}$, and experimented with various combinations. The descriptor sizes ranged from a minimum of 1×512, which is the combined size of ICAT and SCAT descriptors, to a maximum of 1×1024, ensuring efficient search speed. As shown in Figure 7, the most effective performance was observed with a descriptor combining IS-CAT, SR, and IR. Furthermore, the combination of IS-CAT and SR also demonstrated high efficacy. This outcome indicated the significance of SR (i.e., range images encoded with depth values) as a vital 2D representation and form of information. From these graph results, it is evident that important information follows in the order of SR, SB, IR, and IB. The NCLT dataset, which, despite including indoor data, predominantly comprises large-scale outdoor datasets, thereby emphasizing the value of spatial information. Moreover, as mentioned in [10], our results aligned with the observation that, for the NCLT dataset, the range view representation was more effective than the BEV representation.

### 4.4. Place Recognition Performance

This experiment evaluated place recognition in a large-scale environment. To evaluate the NCLT dataset, we performed training on the 8 January 2012 sequence, and evaluation was conducted on the sequences 5 February 2012, 15 June 2012, and 5 April 2013. The evaluation was conducted using average top 1 recall (AR@1), top 5 recall (AR@5), and top 20 recall (AR@20) as metrics. The evaluation results are presented in Table 2. As evident, the proposed model, IS-CAT, exhibited state of the art for a specific sequence. The sequences of 5 February 2012 and 15 June 2012 exhibited the best performance for AR@1 and AR@5; however, SeqOT exhibited the best performance for AR@20. SeqOT generated a descriptor that fused spatial and temporal information based on sequential LiDAR range images. Consequently, it exhibited high performance as it contained more sequences with the increase in the number of candidates. Nevertheless, our model was almost similar to the performance of SeqOT’s AR@20. In the case of the 5 April 2013 sequence, it was observed that CVTNet exhibited the highest performance. It is important to note that, even when spatial information remains consistent over time, the sensitivity of material properties to changes can lead to a slight decrease in performance. This indicates that, while our method effectively utilizes intensity data, it may also be more susceptible to variations in material properties over time.

**Figure 8:**
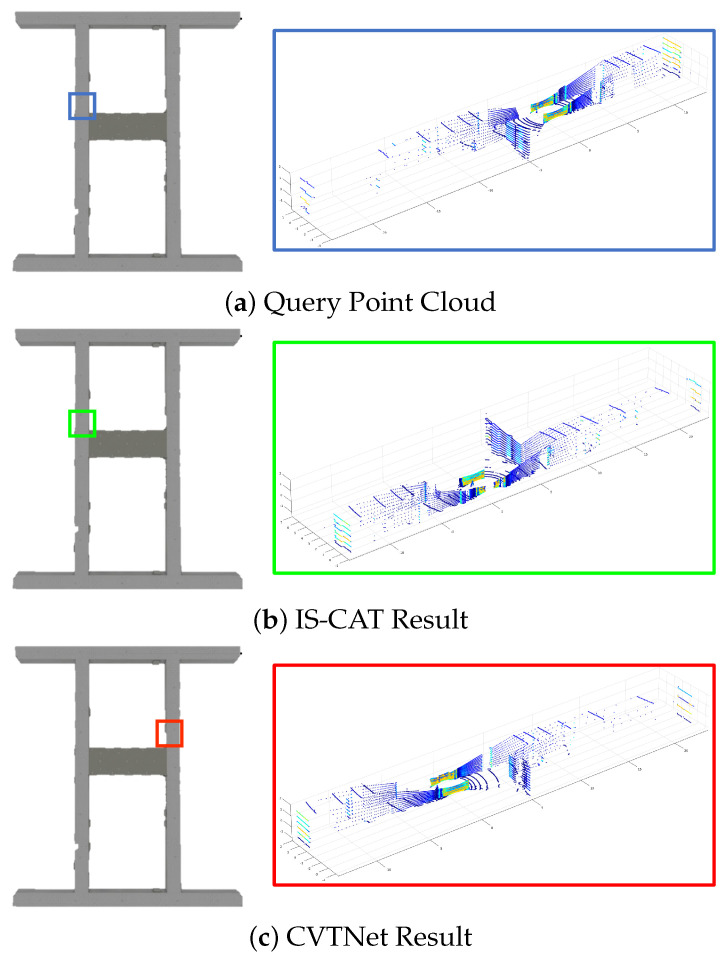
Comparative place recognition outcomes on the Sejong indoor-5F dataset. In this figure, blue represents the query locations, green indicates the correct answers, and red signifies the results of incorrect place recognition. The results highlight the proficiency of IS-CAT in correctly identifying the correct indoor location, in contrast to CVTNet, which, due to its reliance on spatial information, occasionally misidentifies places at symmetrically opposite sites.

We also evaluated the indoor performance using our Sejong indoor-5F dataset. As shown in Figure 5b, the database section represents paths where the AGV navigated the area twice, while the query section shows a single loop on the top floor. The assessment was carried out using models previously trained on the NCLT dataset. We only compared the performance with the existing state-of-the-art model, CVTNet. The results are presented in Table 3. We found that in environments less sensitive to temporal changes, such as indoors, our method significantly outperformed others. As shown in Figure 8, CVTNet struggled to identify the correct locations in spaces with similar structures, because of dealing only with spatial information. In contrast, IS-CAT, by incorporating information about the material properties of surrounding objects, demonstrated markedly improved performance in these environments, even when the spatial structures were similar.

### 4.5. Ablation Study on IS-CAT

**Figure 9:**
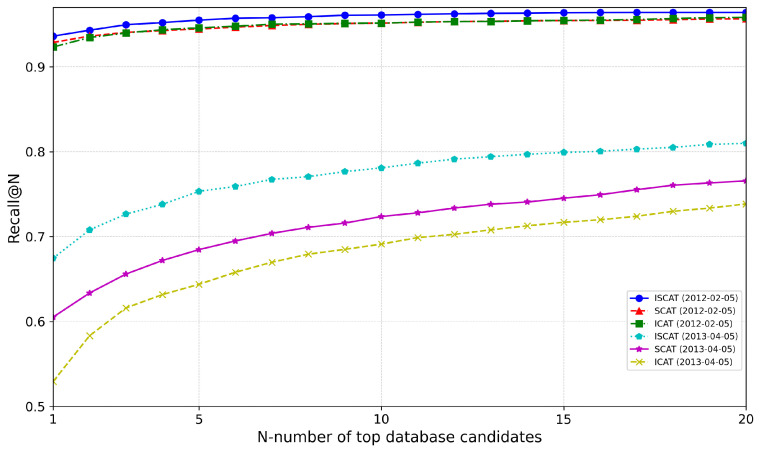
Analysis of ICAT and SCAT modules through the ablation study on the NCLT dataset.

This ablation study further validates the experimental outcomes by inspecting the IS-CAT descriptor into its constituent modules, ICAT and SCAT. We utilized a model trained on the 5 January 2012 sequence on NCLT, and the results for the 5 February 2012 and 5 April 2012 sequences are depicted in Figure 9. For all sequences, we observed IS-CAT’s superior overall performance. The 5 February 2012 sequence shows that ICAT and SCAT have comparable efficacy. It is noted that, while SCAT outperforms at AR@1, ICAT excels at AR@20. The 5 April 2012 sequence, approximately a year later, distinctly demonstrates performance about temporal fluctuations. Here, ICAT and SCAT exhibit significant variances, indicating that spatial data retain their reliability over time, more so than reflective intensity data. These findings underscore the importance of incorporating temporal factors to enhance the IS-CAT model’s accuracy.

### 4.6. Computational Analysis

In this section, we examine our model’s computational complexity and time cost, encompassing both theoretical and empirical perspectives. Initially, the generation of I-S Images depends on the number of data points, *n*, incorporating an efficient sorting mechanism, thus the time complexity remains primarily at O(nlogn). Furthermore, while the cross-attention transformer complexity [33] originally presents an $O(n^{2})$ complexity, where *n* is the token sequence length, the transition to the concatenation phase maintains the same order of magnitude at $O({\left(N\right)}^{2})$, with *N* being the combined token sequence length from both data modalities. Lastly, our search algorithm, leveraging the FAISS [36] library’s WarpSelect algorithm, operates with a complexity of O(klog(l)), where *k* represents the number of nearest neighbors and *l* the length of input data.

To evaluate the runtime efficiency of our model empirically, we conducted a series of live tests on an AGV’s edge device equipped with an Intel i9-12900HK CPU and an RTX 3080 Ti 16GB GPU, utilizing both Python and C++ programming languages. The test dataset used our Sejong indoor-5F dataset. This indoor dataset comprised a total of 2192 sequences, and all measurement times were calculated by considering the average. In Python, our initial experiments focused on generating a 1×1024 descriptor, during which we recorded two primary elements, four image sets, and a 1D descriptor. The average time required to generate four image datasets was approximately 14.32 ms, and the average inference time for the IS-CAT model to produce the descriptor was approximately 15.88 ms. For search speed, utilizing the FAISS library to accelerate the retrieval process, we discovered that identifying the top 20 candidates required an average of 34.58 ms. Consequently, the total time for descriptor generation and retrieval in Python amounted to approximately 64.78 ms. Similarly, when executing the code in C++, the same processes were timed for comparative purposes. The creation of four image datasets required an average of 12.48 ms, whereas the descriptor generation and model inference, enhanced by libtorch, required approximately 3.33 ms. The average time for search operations was 16.78 ms, culminating in a total time of approximately 32.59 ms. These results indicate that our model operates faster than the typical 10–20 Hz operation rates of standard LiDAR systems, demonstrating its suitability for real-time applications in autonomous driving. However, we recognize the need for continuous optimization to further enhance the model’s speed and efficiency in various runtime environments.

### 4.7. 3D SLAM Application

**Figure 10:**
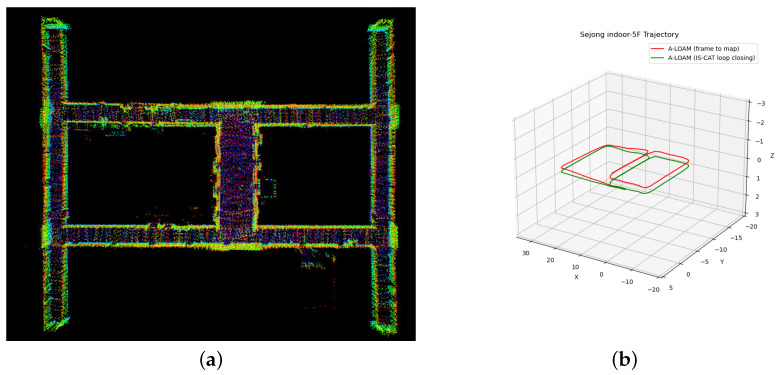
Comparative trajectory analysis using A-LOAM on the Sejong indoor-5F dataset. (**a**) Visualization of Sejong indoor-5F map with 3D SLAM using IS-CAT; (**b**) trajectories of Sejong indoor-5F. The red line of (**b**) shows the A-LOAM (frame to map) outcome, revealing an uncompleted loop closure and the persistence of accumulated drifts. The green line of (**b**) shows the successful loop closure achieved through IS-CAT integration, showcasing the corrected and accurate trajectory.

Inspired by [37], we integrated our IS-CAT model as a loop closure detection module in a 3D LiDAR SLAM system, particularly tested on the Sejong indoor-5F dataset, an indoor environment. Our approach began with the construction of the initial pose-graph using A-LOAM, which is a recognized LiDAR odometry method with only LiDAR data. Upon detecting a loop, a pose-graph was introduced, enabling trajectory optimization through pose-graph optimization with g2o [38]. This emphasized the crucial role of loop closure in 3D SLAM systems. The Sejong indoor-5F dataset, characterized by its indoor settings such as long featureless hallways, is plagued by substantial challenges in the case of feature point detection. As shown in Figure 10, methods relying solely on A-LOAM exhibit suboptimal outcomes. A-LOAM reduces errors present in odometry by using a global map, but it does not entirely eliminate the drift error accumulation. In particular, Figure 10b shows a significant drift error on the z-axis. However, our approach successfully optimized these existing errors by accurately detecting loops, thereby enabling the creation of a precise map and estimating an accurate pose. Our method’s ability to detect loops even in featureless indoor environments was proven by the simultaneous utilization of spatial and intensity information with a variety of views. We have effectively shown its applicability in a SLAM system, proving its efficacy not just in large-scale outdoor environments but also in feature-sparse indoor settings.

## 5. Discussion and Future Work

Our experiments demonstrate that the IS-CAT model excels in place recognition, outperforming current state-of-the-art methods, particularly in indoor settings. By integrating spatial and intensity features from LiDAR data, IS-CAT effectively encapsulates diverse environmental information, proving especially valuable in settings where reflection intensity is crucial. However, several challenges and limitations were identified.

Environmental sensitivity: the model shows heightened sensitivity to environmental changes, such as seasonal variations, which can alter both spatial and intensity features. This was evident in our ablation study, where the model performed exceptionally in consistent environmental conditions but struggled with temporal changes. Addressing this, future iterations of IS-CAT should include adaptive algorithms capable of dynamically updating environmental understanding. This could involve integrating temporal dynamics using time-series LiDAR data, thereby reducing the model’s sensitivity to environmental changes over time.

Intensity data limitations: while specular intensity data enhance place recognition, they also introduce increased susceptibility to external factors like lighting and material properties. This necessitates further research into balancing the impact of reflective intensity against spatial information, aiming for a model that can generalize better across diverse conditions.

Computational complexity: lastly, although the computational demands of the IS-CAT model are currently manageable, several aspects (image generation, model inference, retrieval speed) may pose challenges in real-time applications due to computational complexity. Optimizing these aspects is critical for efficient deployment and ensures the practicality of the model in a variety of applications. Concurrently, we plan to develop algorithms to streamline the model’s computational processes, especially during the cross-attention-to-concatenation phase.

## 6. Conclusions

In this paper, we proposed a novel IS-CAT network, with a variety of views and information for place recognition. Our approach used a cross-attention transformer-to-concatenation method by leveraging multiple views with spatial and intensity information to generate robust descriptors in any environment. We compared the place recognition performance of our method with the state-of-the-art LPR algorithm on the NCLT dataset and Sejong indoor-5F dataset. Notably, on the indoor dataset, Sejong indoor-5F, our method demonstrated satisfactory and robust performance, underscoring its effectiveness in indoor place recognition scenarios. In addition, we applied the proposed method to a real 3D SLAM system with our indoor dataset to correct the accumulated drifts in the robot trajectory through loop closure detection and create an accurate indoor point cloud map. Future work will focus on enhancing the IS-CAT model’s adaptability to environmental variations and optimizing computational efficiency for real-time applications.

## Figures and Tables

**Table 1:** Model Parameters.

Module	Parameters	Value
Transformer	$d_{model}$	128
	$n_{head}$	4
	$d_{ffn}$	1024
Cross-Attention Transformer	$d_{model_{b}}$	256
	$d_{model_{a}}$	512
NetVlad	$d_{r}$	256
	$d_{ri}$	256
	$d_{scat}$	512
	$d_{icat}$	512
	$d_{k}$	64

**Table 2:** Evaluation performance on NCLT dataset. Bold denotes the highest performance in each metric.

Method		5 February 2012			15 June 2012			5 April 2013		
		AR@1	AR@5	AR@20	AR@1	AR@5	AR@20	AR@1	AR@5	AR@20
Hand-Crafted Descriptor	SC [9]	0.767	0.836	0.909	0.581	0.637	0.724	0.418	0.496	0.649
	ISC [18]	0.696	0.839	0.905	0.381	0.514	0.669	0.392	0.457	0.569
	M2DP [24]	0.777	0.803	0.917	0.489	0.557	0.616	0.363	0.455	0.567
	RID [10]	0.790	0.849	0.914	0.597	0.687	0.742	0.473	0.532	0.673
Learning-Based Descriptor	PointNetVLAD [5]	0.746	0.823	0.875	0.612	0.720	0.782	0.449	0.576	0.683
	MinkLoc3D [8]	0.802	0.864	0.926	0.630	0.685	0.774	0.482	0.587	0.685
	OverlapTransformer [13]	0.861	0.899	0.930	0.639	0.697	0.780	0.496	0.603	0.715
	SeqOT [15]	0.917	0.947	**0.968**	0.762	0.844	**0.899**	0.639	0.724	0.826
	FusionVLAD [32]	0.786	0.870	0.922	0.462	0.579	0.702	0.429	0.553	0.667
	CVTNet [16]	0.932	0.946	0.957	0.793	0.835	0.871	**0.780**	**0.826**	**0.869**
	IS-CAT (ours)	**0.936**	**0.954**	0.964	**0.804**	**0.848**	0.885	0.674	0.753	0.813

**Table 3:** Evaluation performance on Sejong indoor-5F dataset.

Method	True Positives	False Positives	AR@1
CVTNet	193	95	0.670
IS-CAT	228	60	0.782

## Data Availability

The data presented in this study are available on request from the corresponding author.
